# Supplementary material for: Electrostatic Engineering of Phosphoketolase Enhances Activity on Small Nonphosphorylated Sugars and Improves Cell-Free ATP Regeneration from Inexpensive C2‑Substrates
Source: J Phys Chem B. 2026 Jul 28;130(31):7791–808. doi: 10.1021/acs.jpcb.6c02050 (PMC13455062; doi:10.1021/acs.jpcb.6c02050)
Supplement: Supplementary file 1 [file jp6c02050_si_001.pdf]

Supporting Information 1

Electrostatic Engineering of Phosphoketolase Enhances Activity on Small Non-phosphorylated Sugars and Improves Cell-free ATP Regeneration from Inexpensive C_2-substrates

Franziska Kraußer[1], Christopher M. Topham[2], Kenny Rabe[1], Jan-Ole Kundoch[3], Daniel Ohde[3], Andreas Liese[3], Thomas Walther[1*]

[1]Chair of Bioprocess Engineering, Institute of Natural Materials Technology, TU Dresden,

Bergstraße 120, 01062 Dresden, Germany

[2] Molecular Forces Consulting, 24 Avenue Jacques Besse, 81500 Lavaur, France

[3] Institute of Technical Biocatalysis, Hamburg University of Technology, Denickestr. 15,

21073 Hamburg, Germany

***Email:** thomas_walther@tu-dresden.de

Phosphoketolase reaction mechanism on GA and ERU

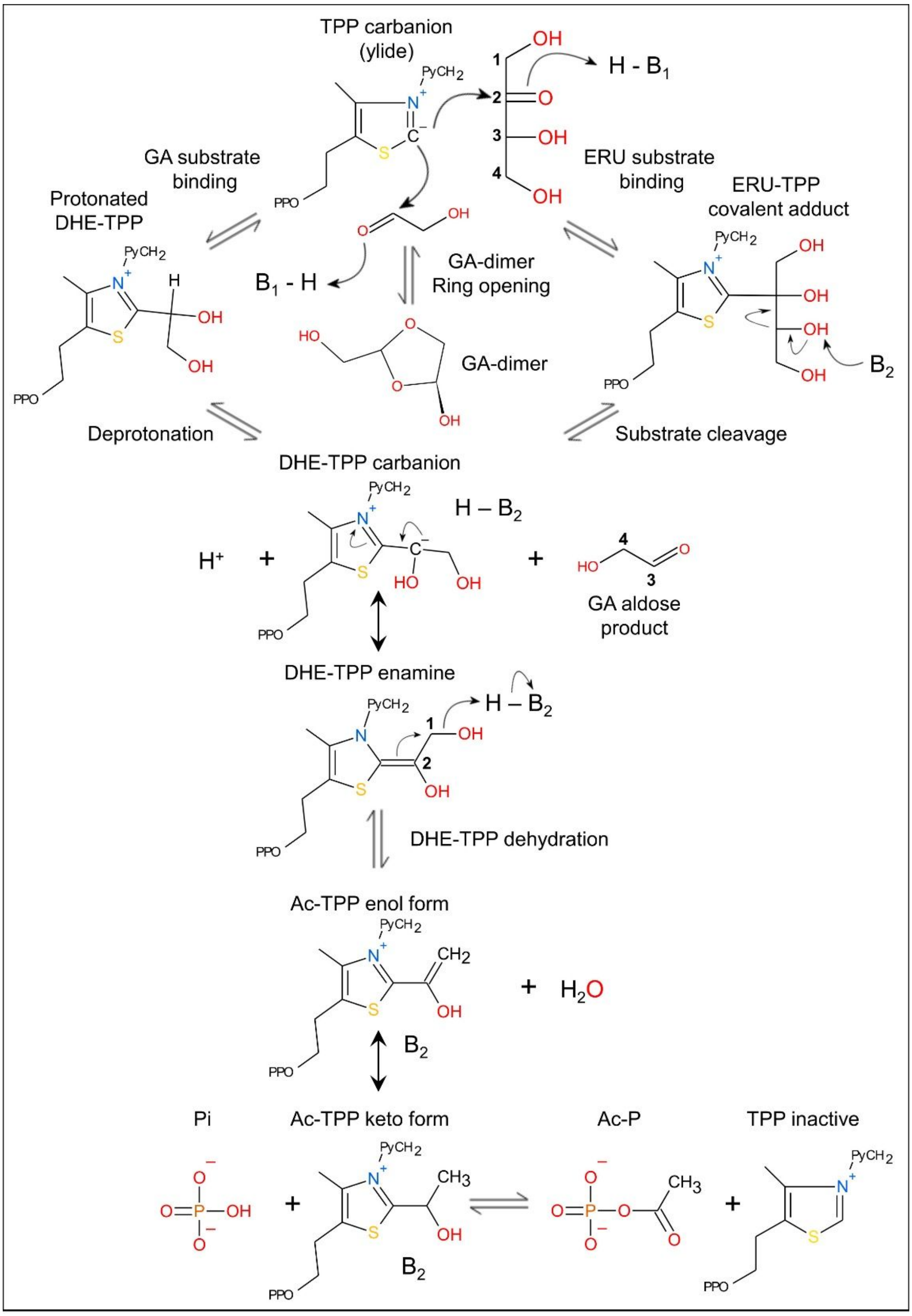

Scheme S1 Phosphoketolase reaction mechanisms on glycolaldehyde and D-erythrulose. Key reaction (resonance) intermediates and catalytic steps implicated in alternative non-phosphorylated substrate-dependent mechanisms[1–7] are indicated. Abbreviations: GA, glycolaldehyde; GA-dimer, 2-hydroxymethyl-4-hydroxy-1,3-dioxolane; ERU, D-erythrulose; TPP, thiamine pyrophosphate; Py, aminopyrimidine; PPO, diphosphate; DHE-, dihydroxyethyl; Ac-, acetyl; Ac-P, acetyl phosphate; Pi, phosphate. Attributed PKT catalytic base residues in the literature: B_1, H553; B_2, H64, H320, H553 (Bad.F6Pkt sequence numbering). Participation of H64 in enzyme-catalysed ring opening of the cyclic GA dimer for *in situ* generation of the monomeric GA species can be inferred from hydrogen bonding interactions with the GA dimer in the modelled complex of Bad.F6Pkt and the Mg-TPP cofactor (Figure 2B).

Glycolaldehyde cyclic dimer structure and force field parameters

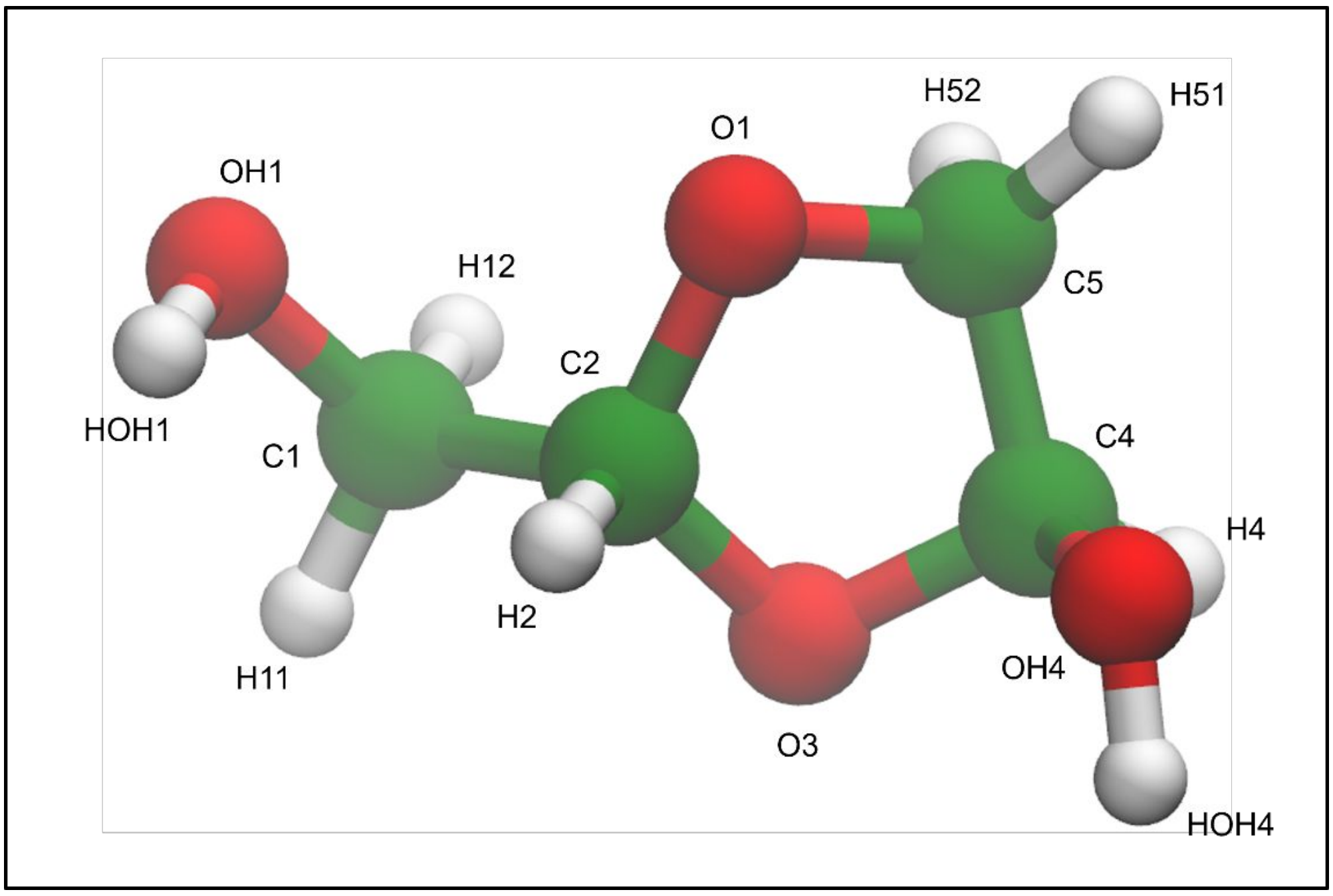

Figure S1 2-Hydroxymethyl-4-hydroxy-1,3-dioxolane glycolaldehyde cyclic dimer structure.

Table S1 Partial charges and GAFF atom types for 2-hydroxymethyl-4-hydroxy-1,3-dioxolane. RESP partial charges[8] were obtained from fitting to the HF/6-31G* quantum chemical electrostatic potential of the fully geometry-optimised structure. Atom names are shown in Supporting Information Figure S1.

Atom Name	Partial Charge	GAFF Atom Type
C1	0.0575	c3
C2	0.3147	c3
C4	0.4906	c3
C5	0.0662	c3
O1	-0.3952	os
OH1	-0.6468	oh
O3	-0.5095	os
OH4	-0.7181	oh
H11	0.0968	h1
H12	0.0968	h1
HOH1	0.4219	ho
H2	0.0899	h2
H4	0.0255	h2
HOH4	0.4579	ho
H51	0.0759	h1
H52	0.0759	h1

Structural anatomy of the phosphoketolase mobile QN-loop

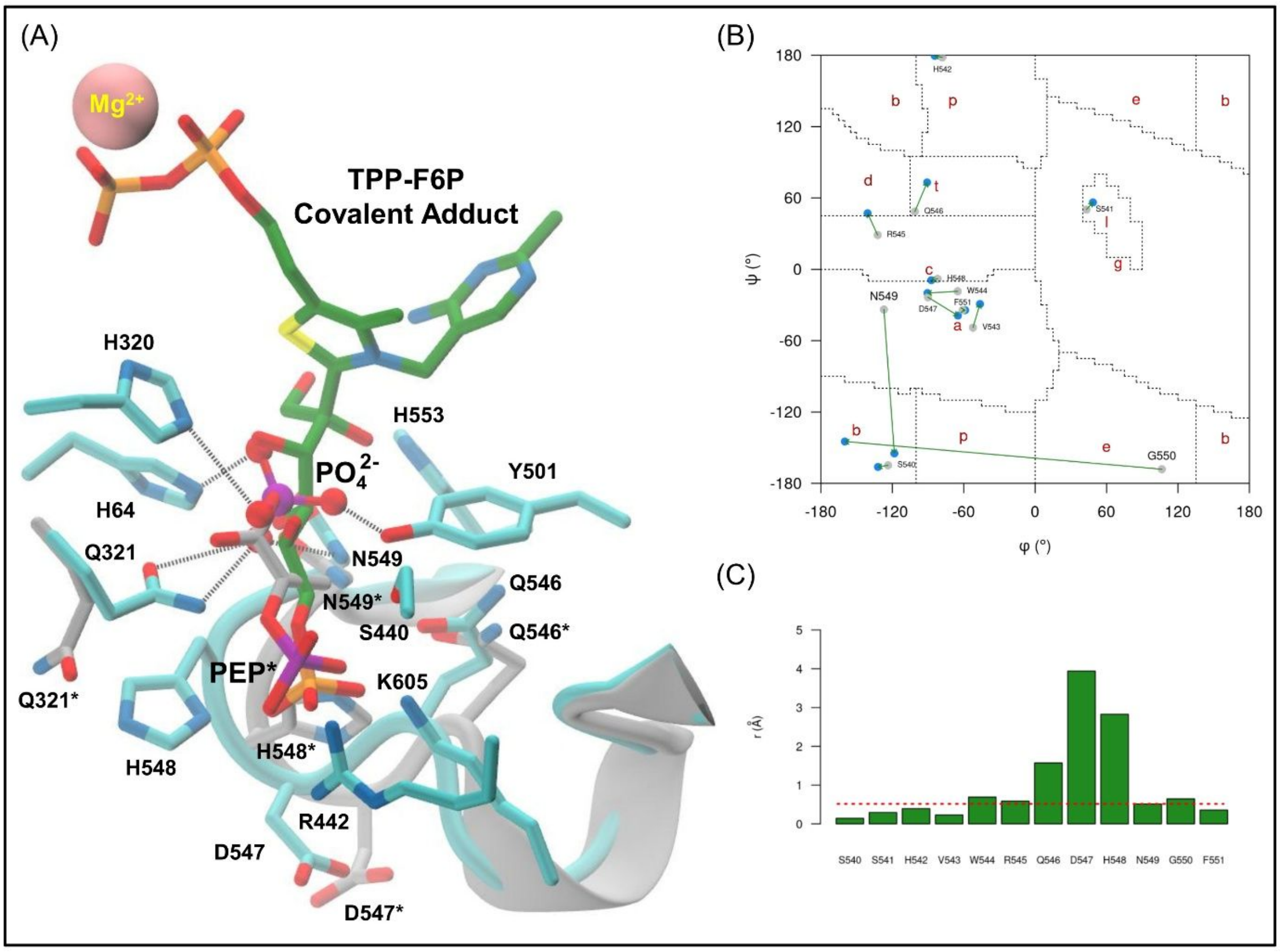

Figure S2 Open and closed QN-loop conformers. (A) Modelled Bad.F6Pkt complex with TPP frutose-6-P overlaid with X-ray structures of *Bifidobacterium longum* PKT-phophoenolpyruvate (PEP) and *Bifidobacterium breve* PKT- phosphate ion complexes. The TPP-fructose-6-P (T6F) covalent adduct and selected Bad.F6Pkt side-chains in the modelled complex [9] comprising a closed Q546-N549 loop are shown in stick representation with green and cyan-coloured carbon atoms, respectively. T6F phosphorus atoms are coloured orange, and the Mg^{2+} ion bound to the TPP pyrophosphate moiety is depicted as a pink-coloured solid van der Waals sphere. Carbon atoms in protein side-chain and PEP inhibitor (* annotated) residues in the *Bifidobacterium longum* open QN-loop PKT complex overlay (PDB code 7c81,[10]) are coloured grey. PKT QN loop closure is accompanied by a change in Q321 side-chain rotamer class [11] from *tm-30* to *tp-100*. Hydrogen bonds between Bad.F6Pkt side-chains in the inorganic phosphate substrate binding site and the overlaid PO_4^{2-} ion (CPK representation) from the *Bifidobacterium breve* closed QN-loop PKT complex (PDB code 3ahf,[6]) are shown as black dashed-line vectors. Phosphorus atoms in the PEP and PO_4^{2-} ion overlays are coloured purple. Other atom types are coloured according to chemical element: oxygen – red, nitrogen – blue, sulphur – yellow. **(B) Ramachandran plot of residue main-chain dihedral angles in structurally variable region comprising Q546-N549 loop.** Plotted dihedral angle values correspond to residue positions in the S540 to F551 fragment connecting β-sheet secondary structural elements in *Bifidobacterium breve* (closed conformation, blue filled circles) and *Bifidobacterium longum* (open conformation, grey filled circles) phosphoketolases, respectively abstracted from 3ahi[6] and 7c8i[10] PDB X-ray crystallographic data sets. Green arrow vectors denote open-to-closed QN-loop residue conformational transitions. Broken black lines define the division of ϕ/ψ dihedral angle space into nine conformational zones with local population maxima in structurally variable regions of experimentally determined protein structures [12] **(C) QN-loop conformer residue distance profile.** C^{α}-C^{α} distance profile obtained from POLYFIT[13] superposition of 3ahi and 7c8i co-ordinate data sets. The red dashed reference line corresponds to an RMSD of 0.52 Å over 802 structurally aligned positions.

Protein electrostatics calculations

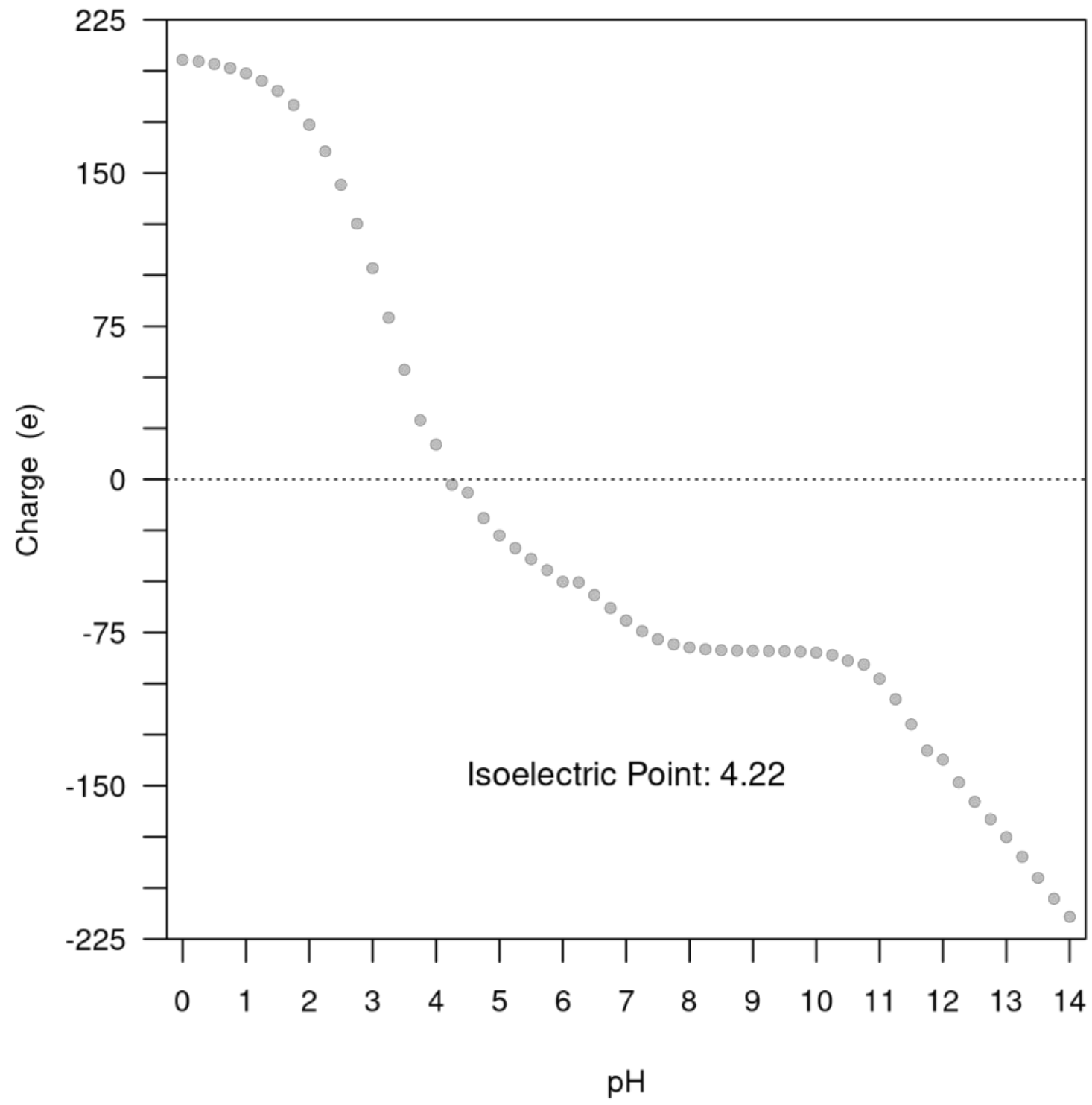

Figure S3 Simulated titration curve for wild-type Bad.F6Pkt. Ionisable residue pK_as were calculated using DelPhiPKa[14,15] at pH intervals of 0.25 at an ionic strength of 0.15 mM as described in Methods. Each monomeric subunit carries a net charge of -36 at pH 7.

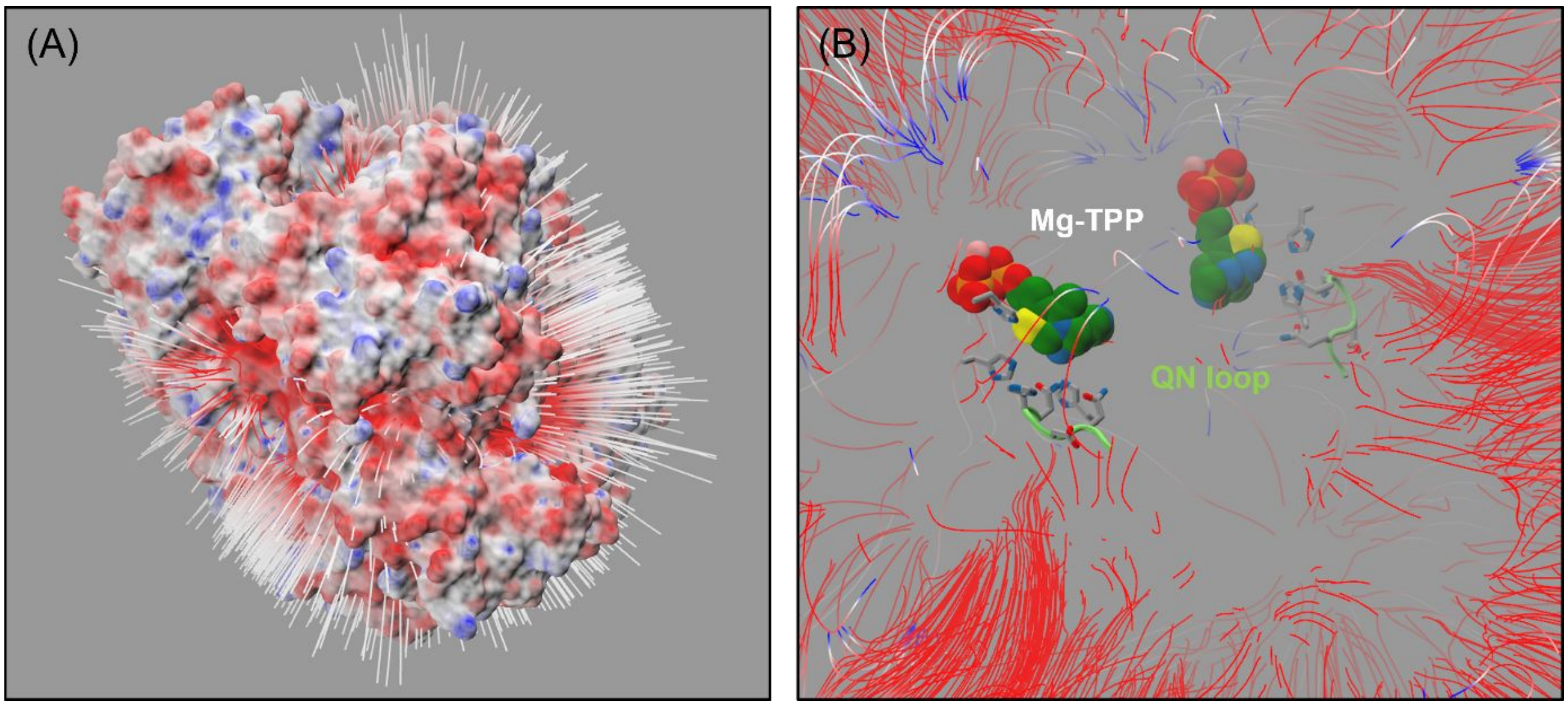

Figure S4 Electrostatic field in wild-type Bad.F6Pkt. Field lines are coloured according to electrostatic potential, with starting points in blue (positive potential) and end points in red (negative potential). **(A) View from protein exterior.** Electrostatic potential mapped onto the solvent-excluded surface is colour scaled at ±10 kT/e thresholds. **(B) Protein interior.** TPP cofactors and histidine (64, 320 and 553) residue side-chains in the catalytic centres of the dimeric enzyme molecule in the same orientation are respectively shown as van der Waals sphere and stick-form representations. The mainchain backbones of the QN (Q546, D547, H548, N549) active-site loops are highlighted as lime green coloured cartoon tubes, and the loop side-chains depicted as sticks with grey coloured carbon atoms.

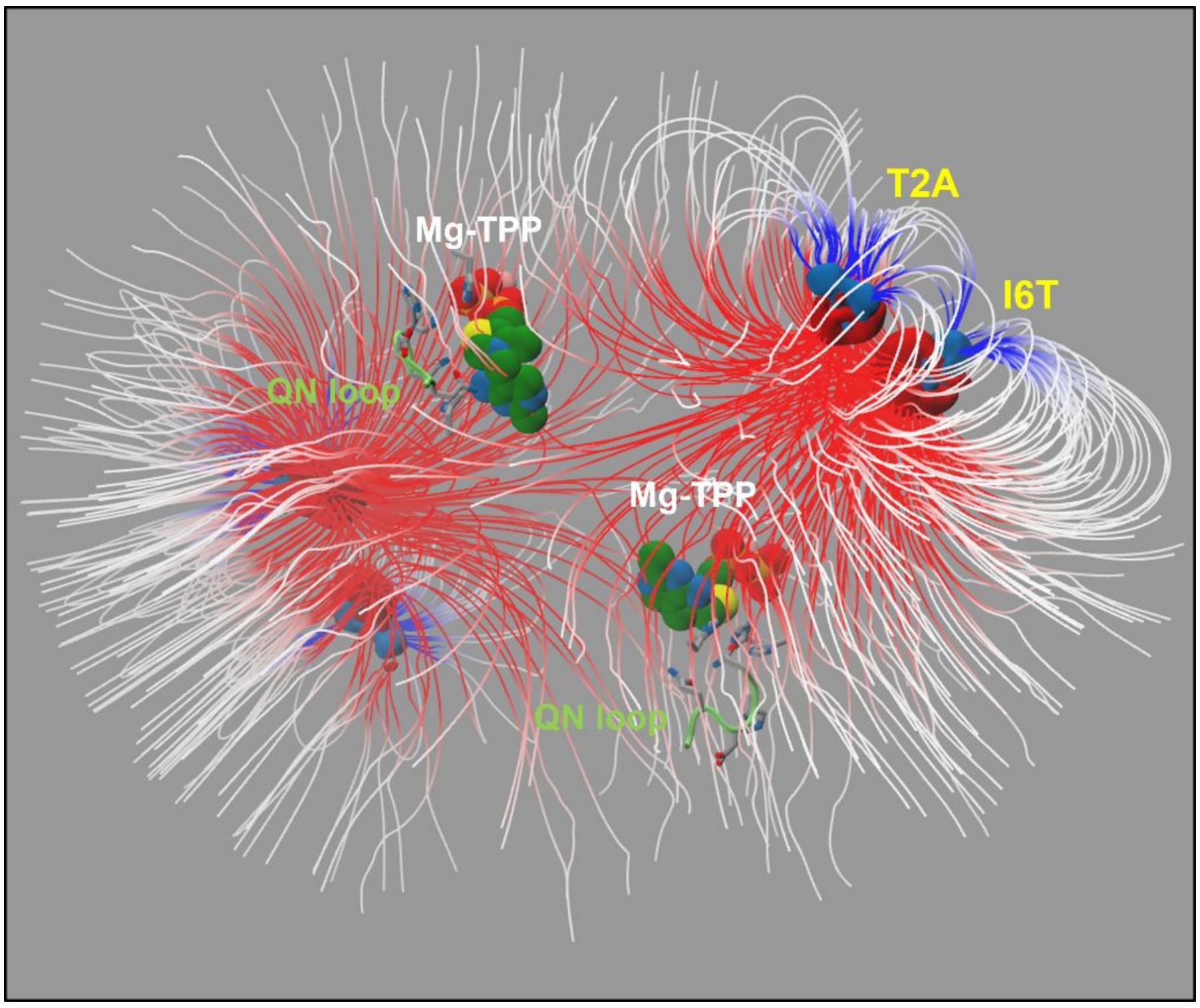

Figure S5 Electrostatic potential difference grid map for Bad.F6Pkt T2A:I6T double mutant. Difference electrostatic potential ($\Delta\phi(\mathbf{r})$), calculated relative to wild-type Bad.F6Pkt, is displayed as solid rendered (±2 kT/e) contoured isosurfaces, respectively coloured in blue or red for positive or negative $\Delta\phi(\mathbf{r})$. Electrostatic field lines are coloured according to the electrostatic difference potential. The two TPP cofactor molecules and their bound Mg^{2+} ions in the enzyme dimer are shown as van der Waals spheres, coloured according to element type: carbon, green; nitrogen, blue; oxygen, red; sulphur, yellow; phosphorus, orange; magnesium, pink. Active-site histidine (64, 320 and 553) and (Q546, D547, H548, N549) QN loop residue side-chains are shown as in stick-form with grey-coloured carbons. The QN loop mainchains are depicted in cartoon form as lime green coloured tubes.

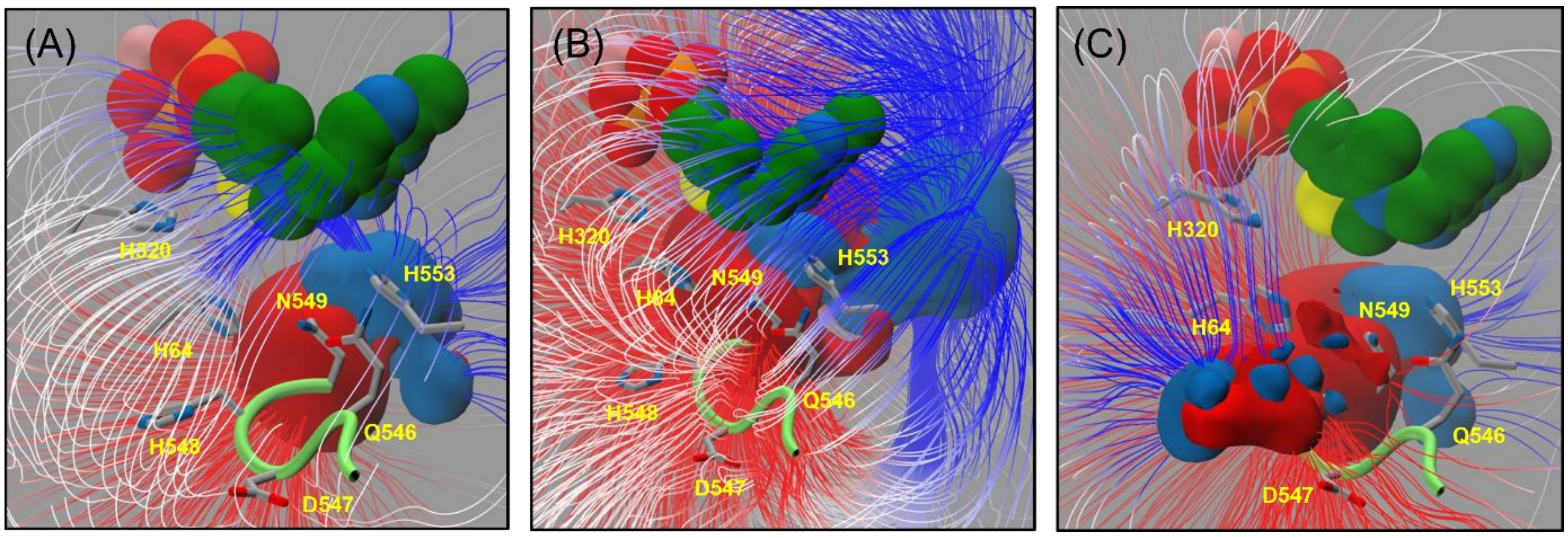

Figure S6 Electrostatic potential difference grid maps for Bad.F6Pkt for H142N mutation carrying variants. (A) H142N **(B)** H142N:E153D. **(C)** H142N:H548Y. Difference electrostatic potential (Δϕ(**r**)), calculated relative to wild-type Bad.F6Pkt, is shown as solid rendered ±4 kT/e contoured isosurfaces, coloured in blue or red for positive or negative Δϕ(**r**), respectively. Electrostatic field lines directed away from positive charge difference sources are coloured according to the electrostatic difference potential due to mutation. The Mg-TPP cofactor is shown as van der Waals spheres with green coloured carbon atoms. Active-site histidine (64, 320 and 553) and QN loop (Q546, D547, H548, N549) residue side-chains are shown as in stick-form with grey-coloured carbons. The QN loop mainchain is depicted in cartoon form as a lime green tube.

Table S2. Computed residue pK_a values in Bad.F6Pkt N-terminal and Asp26 region mutants. Calculations at ionisable residue positions were carried on modelled wild-type and mutant Bad.F6Pkt complexes with Mg-TPP using DelPhiPKa. Tabulated predicted pK_a values at selected positions are averages over both chains in the dimeric enzyme. For clarity, only induced pK_a shifts relative to the wild type enzyme (ΔpK_a) of ≥ ±0.1 in magnitude are indicated below in parentheses. RSA, side-chain residue solvent accessible surface area relative to that in corresponding extended conformation Ala-XXX-Ala model reference tri-peptide structure [16]. NA, not applicable.

Bad.F6Pkt Residue Position	RSA (%)	Wild Type	H256P	H256Y	H260Y	H256Y: H260Y	T2A: I6T	T2A: I6T: H260Y
D26	13	1.97	2.30 (+0.33)	2.29 (+0.32)	2.42 (+0.45)	2.76 (+0.79)	1.97	2.50 (+0.53)
H59	26	5.55	5.55	5.55	5.55	5.55	5.55	5.55
H64	2	5.08	5.09	5.09	5.09	5.09	5.08	5.04
H97	0	6.09	6.09	6.09	6.10	6.10	6.09	6.05
H142	2	4.90	4.91	4.91	4.91	4.91	4.90	4.84
E153	21	2.23	2.23	2.23	2.23	2.23	2.23	2.22
H256	46	6.08	NA	NA	6.22 (+0.14)	NA	6.08	6.20 (+0.12)
H260	0	6.38	6.55 (+0.17)	6.54 (+0.16)	NA	NA	6.38	NA
K300	0	11.86	11.86	11.86	11.86	11.78	11.86	11.88
H320	2	4.64	4.65	4.65	4.65	4.66	4.64	4.71
E368	55	4.00	4.00	4.00	4.00	4.00	3.98	3.97
E384	67	3.98	3.98	3.98	3.98	3.98	3.97	3.99
D428	47	3.43	3.43	3.43	3.43	3.43	3.45	3.41
E437	1	2.23	2.24	2.24	2.24	2.24	2.24	2.24
R442	31	13.16	13.16	13.13	13.17	13.16	13.16	13.17
E479	1	2.58	2.58	2.50	2.58	2.58	2.58	2.58
D547	26	1.19	1.19	1.19	1.19	1.19	1.18	1.18
H548	29	5.52	5.52	5.52	5.52	5.52	5.52	5.53
H553	0	4.93	4.93	4.93	4.93	4.93	4.93	4.96
K605	19	10.76	10.76	10.71	10.76	10.76	10.76	10.79

Table S3 Computed residue pK_a values in Bad.F6Pkt QN loop and catalytic-centre mutants. Calculations at ionisable residue positions were carried on modelled wild-type and mutant Bad.F6Pkt complexes with Mg-TPP using DelPhiPKa. Tabulated predicted pK_a values at selected positions are averages over both chains in the dimeric enzyme. For clarity, only Induced pK_a shifts relative to the wild type enzyme (ΔpK_a) of ≥ ±0.1 in magnitude are indicated below in parentheses. RSA, side-chain residue solvent accessible surface area relative to that in corresponding extended conformation Ala-XXX-Ala model reference tri-peptide structure [16]. NA, not applicable.

Bad.F6Pkt Residue Position	RSA (%)	Wild Type	E153D	H142N:E153Q	H142N:H548Y	D547E	D547E:H548Y
D26	13	1.97	1.97	1.97	1.98	1.97	1.97
E55	80	3.94	3.94	3.94	3.95	3.94	3.94
D56	14	3.09	3.09	3.12	3.13	3.09	3.12
H59	26	5.55	5.55	5.56	5.64	5.58	5.64
H64	2	5.08	5.09	5.29 (+0.21)	5.69 (+0.61)	5.07	5.39 (+0.31)
H97	0	6.09	6.12	6.07	6.12	6.09	6.09
H142	2	4.90	5.18 (+0.28)	NA	NA	4.89	4.94
E153	21	2.23	1.55 (-0.68)	NA	2.45 (+0.22)	2.23	2.25
H256	46	6.08	6.08	6.08	6.08	6.08	6.08
H260	0	6.38	6.38	6.38	6.42	6.38	6.38
K300	0	11.86	11.87	11.82	11.85	11.86	11.86
H320	2	4.64	4.64	4.66	4.78 (+0.14)	4.63	4.74 (+0.10)
E437	1	2.23	2.24	2.30	2.37 (+0.14)	2.24	2.29
R442	31	13.16	13.16	13.15	13.15	13.21	13.17
E479	1	2.58	2.58	2.63	2.64	2.58	2.59
D547	26	1.19	1.19	1.22	1.47 (+0.28)	1.98 (+0.79)	2.25 (+1.06)
H548	29	5.52	5.51	5.52	NA	5.55	NA
H553	0	4.93	4.89	4.92	5.26 (+0.34)	4.87	4.97
K605	19	10.76	10.76	10.73	10.80	10.77	10.80

Table S4 Computed residue pK_a values in Bad.F6Pkt substrate binding channel mutants at positions D56 and N549. Calculations at ionisable residue positions were carried on modelled wild-type and mutant Bad.F6Pkt complexes with Mg-TPP using DelPhiPKa. Tabulated predicted pK_a values at selected positions are averages over both chains in the dimeric enzyme. For clarity, only Induced pK_a shifts relative to the wild type enzyme (ΔpK_a) of ≥ ±0.1 in magnitude are indicated below in parentheses. RSA, side-chain residue solvent accessible surface area relative to that in corresponding extended conformation Ala-XXX-Ala model reference tri-peptide structure [16]. NA, not applicable.

Bad.F6Pkt Residue Position	RSA (%)	Wild Type	D56N	N549D	N549C	N549S	N549V
D26	13	1.97	1.97	1.97	1.97	1.97	1.97
E55	80	3.94	3.82 (-0.12)	3.94	3.94	3.94	3.94
D56	14	3.09	NA	3.09	3.09	3.10	3.09
H59	26	5.55	5.51	5.58	5.55	5.55	5.55
H64	2	5.08	5.08	6.04 (+0.96)	5.15	4.93 (-0.15)	4.93 (-0.15)
H97	0	6.09	6.09	6.10	6.06	6.07	6.06
H142	2	4.90	4.90	5.21 (+0.31)	4.71 (-0.19)	4.74 (-0.16)	4.65 (-0.25)
E153	21	2.23	2.23	2.30	2.22	2.23	2.23
H256	46	6.08	6.07	6.08	6.08	6.08	6.08
H260	0	6.38	6.38	6.38	6.38	6.38	6.38
K300	0	11.86	11.86	11.95	11.85	11.85	11.84
H320	2	4.64	4.64	4.77 (+0.13)	4.64	4.65	4.64
E437	1	2.23	2.24	2.42 (+0.18)	2.25	2.25	2.25
R442	31	13.16	13.16	13.30 (+0.14)	13.17	13.16	13.16
E479	1	2.58	2.58	2.58	2.58	2.58	2.57
D547	26	1.19	1.19	1.27	1.20	1.20	1.20
H548	29	5.52	5.53	5.67 (+0.15)	5.55	5.52	5.54
N549	5	NA	NA	0.36	NA	NA	NA
H553	0	4.93	4.93	6.10 (+1.17)	4.76 (-0.17)	5.12 (+0.19)	4.87
K605	19	10.76	10.76	11.04 (+0.28)	10.81	10.80	10.80

Multiple sequence alignment analysis

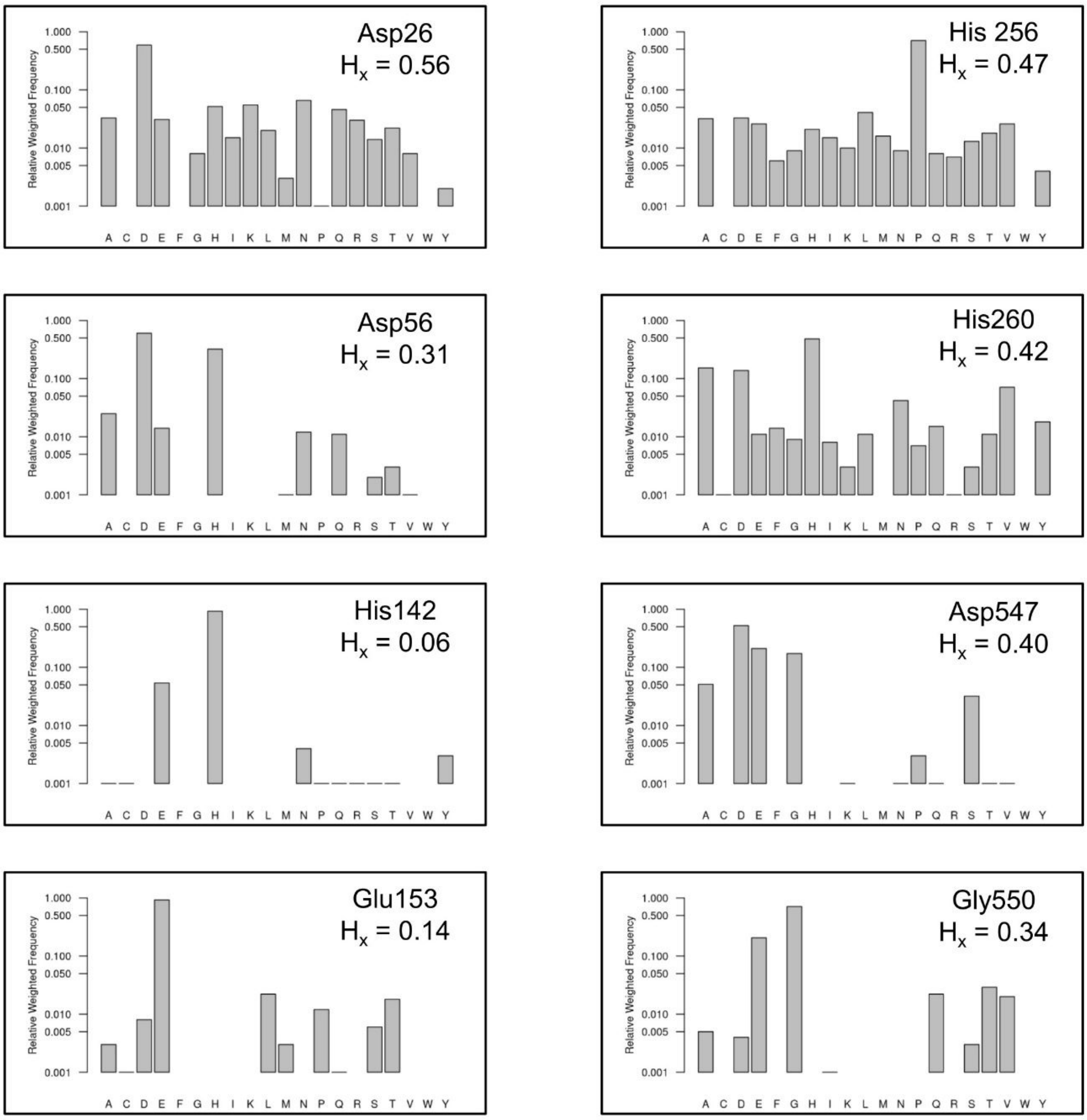

Figure S7 MSA sequence-weighted residue frequency profiles at residue positions in Bad.F6Pkt.

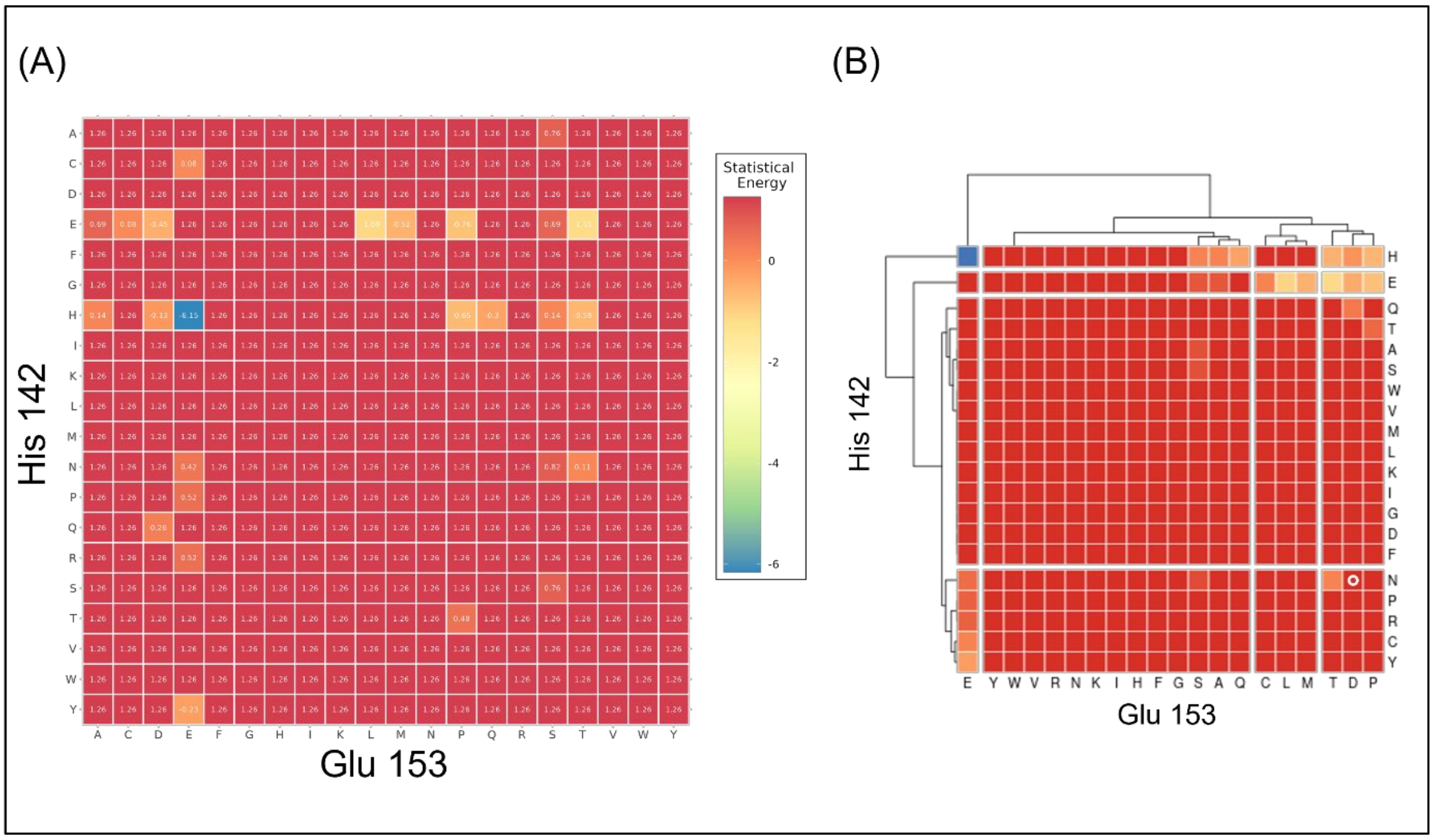

Figure S8 Residue-pair statistical correlation energy heat maps for positions H142 and E153 in Bad.F6Pkt. (A) Matrix heatmap annotated with statistical energies, calculated from sequence weighted observed residue type counts in a multiple sequence alignment as described in Methods using SEQUESTER [17]. **(B)** Hierarchical clustered heat map. The H142N:E153D double mutant is highlighted by the white circle.

Plasmids

Table S5 Plasmids used in this study.

Plasmid	Short description	Source
pET-28a(+)	*ori f1*, Kan^R, T7 promoter	Novagen™
pET28a_*Bad.f6pkt*	pET-28a(+) carrying *f6pkt* gene from *Bifidobacterium adolescentis* ATCC 15703	[9]
pET28a_*Bad.f6pkt* H142N	pET-28a(+) carrying $f6pkt_{H142N}$ gene from *Bifidobacterium adolescentis* ATCC 15703	[9]
pET28a_*Bad.f6pkt* H548N	pET-28a(+) carrying $f6pkt_{H548N}$ gene from *Bifidobacterium adolescentis* ATCC 15703	[9]
pET28a_*Bad.f6pkt* H548Y	pET-28a(+) carrying $f6pkt_{H548Y}$ gene from *Bifidobacterium adolescentis* ATCC 15703	[9]
pET28a_*Bad.f6pkt* N549D	pET-28a(+) carrying $f6pkt_{N549D}$ gene from *Bifidobacterium adolescentis* ATCC 15703	[9]
pET28a_*Bad.f6pkt* N549C	pET-28a(+) carrying $f6pkt_{N549C}$ gene from *Bifidobacterium adolescentis* ATCC 15703	[9]
pET28a_*Bad.f6pkt* N549S	pET-28a(+) carrying $f6pkt_{N549S}$ gene from *Bifidobacterium adolescentis* ATCC 15703	[9]
pET28a_*Bad.f6pkt* N549V	pET-28a(+) carrying $f6pkt_{N549V}$ gene from *Bifidobacterium adolescentis* ATCC 15703	[9]
pET28a_*Ec.fsaA* L107Y:A129G	pET-28a(+) carrying $fsaA_{L107Y:A129G}$ gene from *Escherichia coli* K-12 MG1655	Lab stock
pET28a_*Ps.lrhi*	pET-28a(+) carrying *lrhi* gene from *Pseudomonas stutzerii*	[9]
pET28a_*Gg.der*	pET-28a(+) carrying *der* gene from *Gallus gallus*	[9]
pET28a_*Gs.ackA*	pET-28a(+) carrying *ackA* gene from *Geobacillus stearothermophilus* DSM 22	[9]
pET28a_*Go.gox0313*	pET-28a(+) carrying the codon-optimized *gox0313* gene from *Gluconobacter oxydans* DSM 2003	Lab stock
pET28a_*Ms.nox*	pET-28a(+) carrying the *nox* gene from *Methanobrevibacter smithii* ATCC 35061	Lab stock
pET28a_*Cs.glpK*	pET-28a(+) carrying *glpK* gene from *Cellulomonas sp.* NT3060	[9]

Additional experimental results

Beneficial Bad.F6Pkt variant H548Y identified from screening of selected active site positions

In order to identify an active site-variant with improved affinity for GA, we screened a total of 20 variants in bound TPP-F6P adduct contacting positions Q321, S541, H548 and K605 of Bad.F6Pkt previously found to improve activity on the non-phosphorylated substrate D-fructose[9]. The majority of these active-site variants manifested a significantly impaired GA activity (Figure S9). In addition to the H548N mutation described previously[9], only the H548Y variant exhibited a significant increase in activity of 21 ± 8 % compared to the wild type enzyme. Therefore, the kinetic parameters of the H548Y Bad.F6Pkt variant were analyzed on GA and ERU (Table 2). With a K_M value of 43.4 ± 3.5 mM for the C_2 aldehyde and 26.9 ± 1.9 mM for the C_4 sugar, the H548Y mutation significantly enhanced the apparent affinity for both substrates in comparison to the wild-type and the H548N variant (no substrate saturation up to 300 mM of GA[9]). The k_{cat}/K_M of the H548Y mutant was determined to be 14.8 ± 1.0 M^{-1} s^{-1} for GA. Compared to H548N, an increase of 66 % in catalytic efficiency for ERU was observed, representing a 2.7-fold increase in comparison to the wild-type enzyme (Table 2, Figure 5).

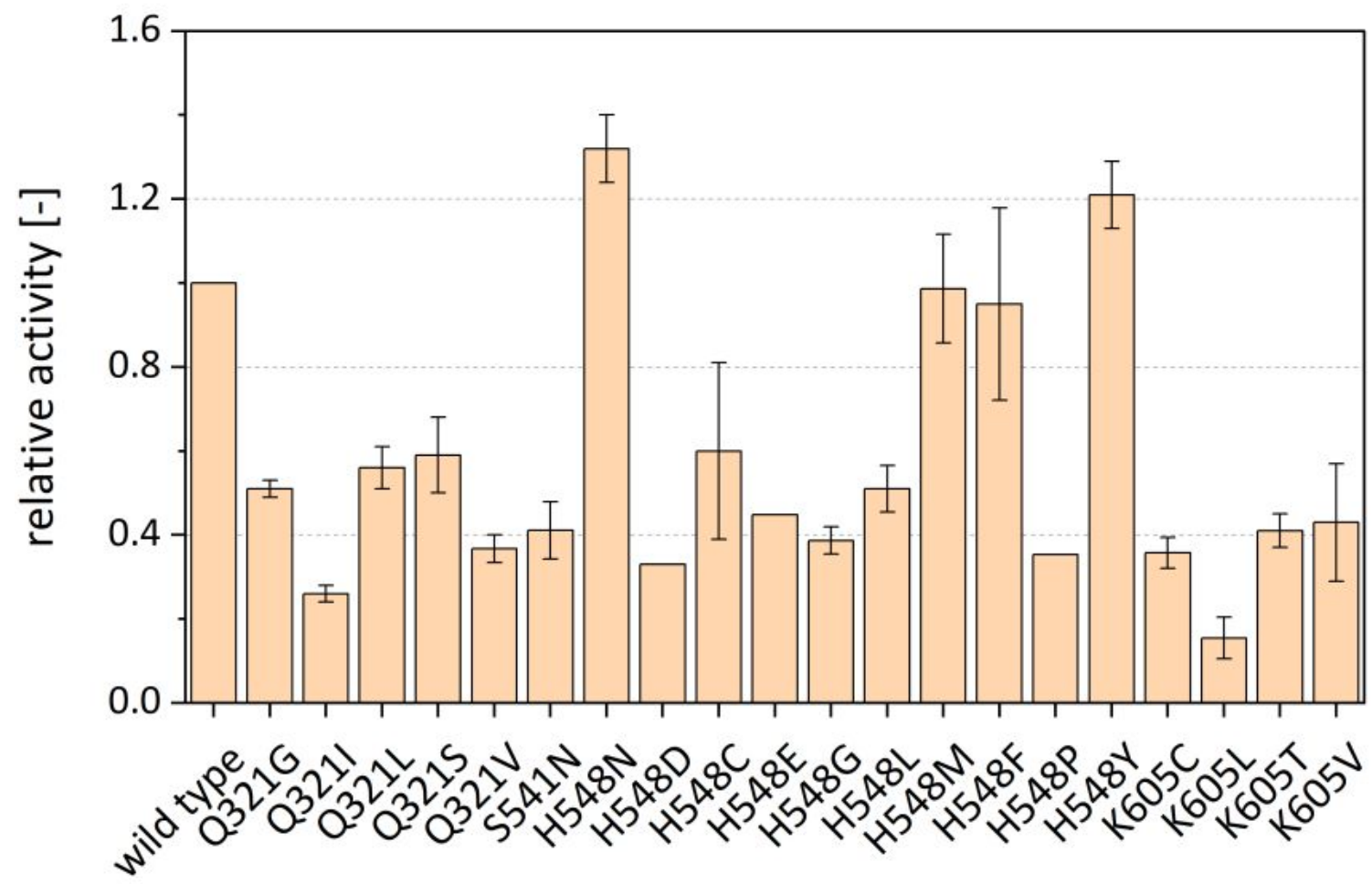

Figure S9 Relative GA activity of Bad.F6Pkt active site single mutants. The selection of mutations was based on their reported positive impact on activity towards the non-phosphorylated substrate D-fructose[9]. Beneficial variants, identified from screening of single mutation libraries, were produced in shake flasks, purified and their specific activity determined at pH 6.5, 37 °C in the presence of 200 mM GA. Relative activity is expressed as the ratio of the specific activities of the mutant and wild-type enzyme. Error bars indicate deviation of the mean (n ≥ 2).

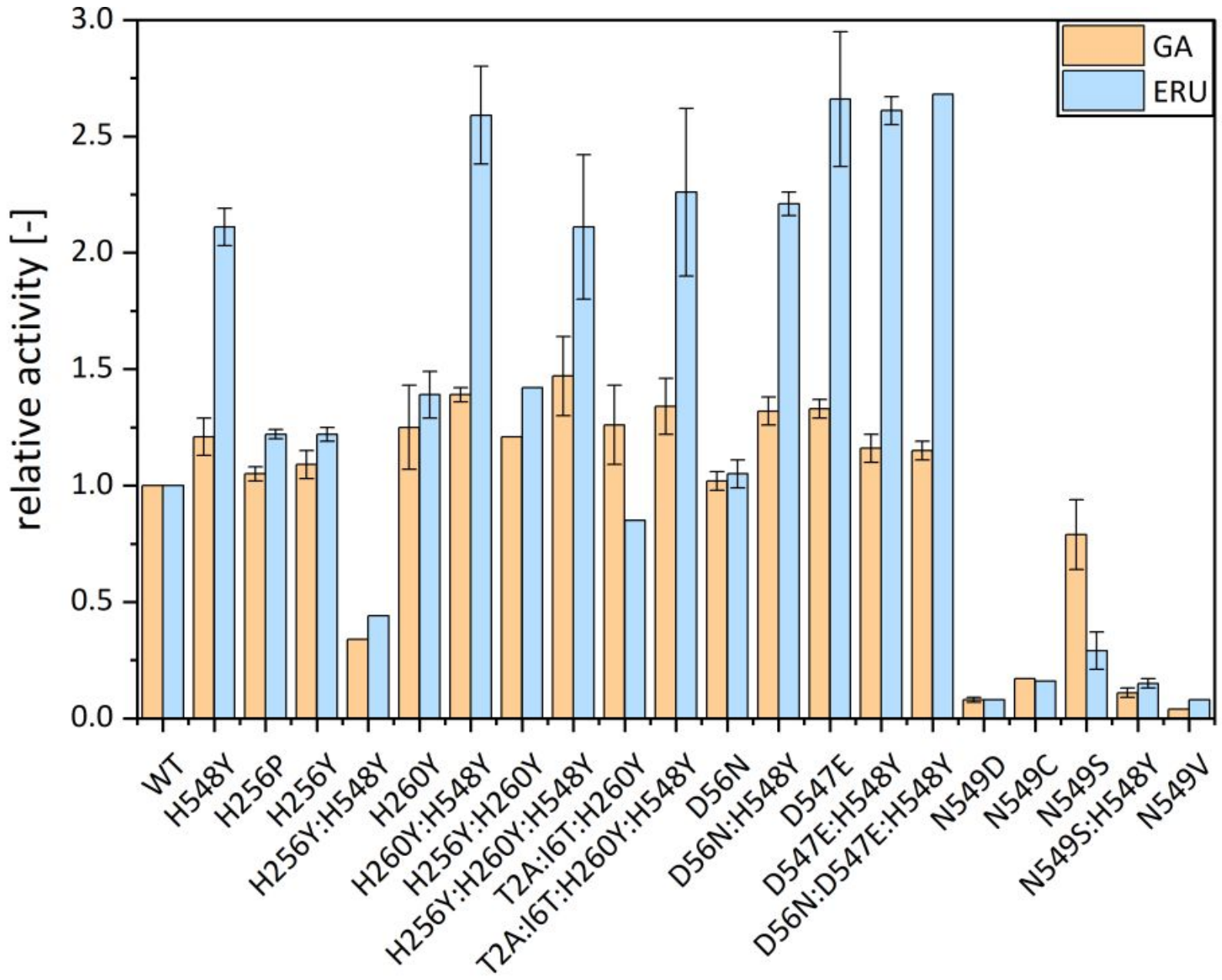

Figure S10 Relative activity on D-erythrulose (ERU) and glycolaldehyde (GA) of Bad.F6Pkt variants carrying electrostatic mutations. WT – wild type. Experiments were carried out at pH 6.5, 37 °C in the presence of 50 mM inorganic phosphate and 25 mM ERU or 200 mM GA. Relative activity is expressed as the ratio of the specific activities of the mutant and wild-type enzyme. Error bars indicate deviation of the mean (n ≥ 2).

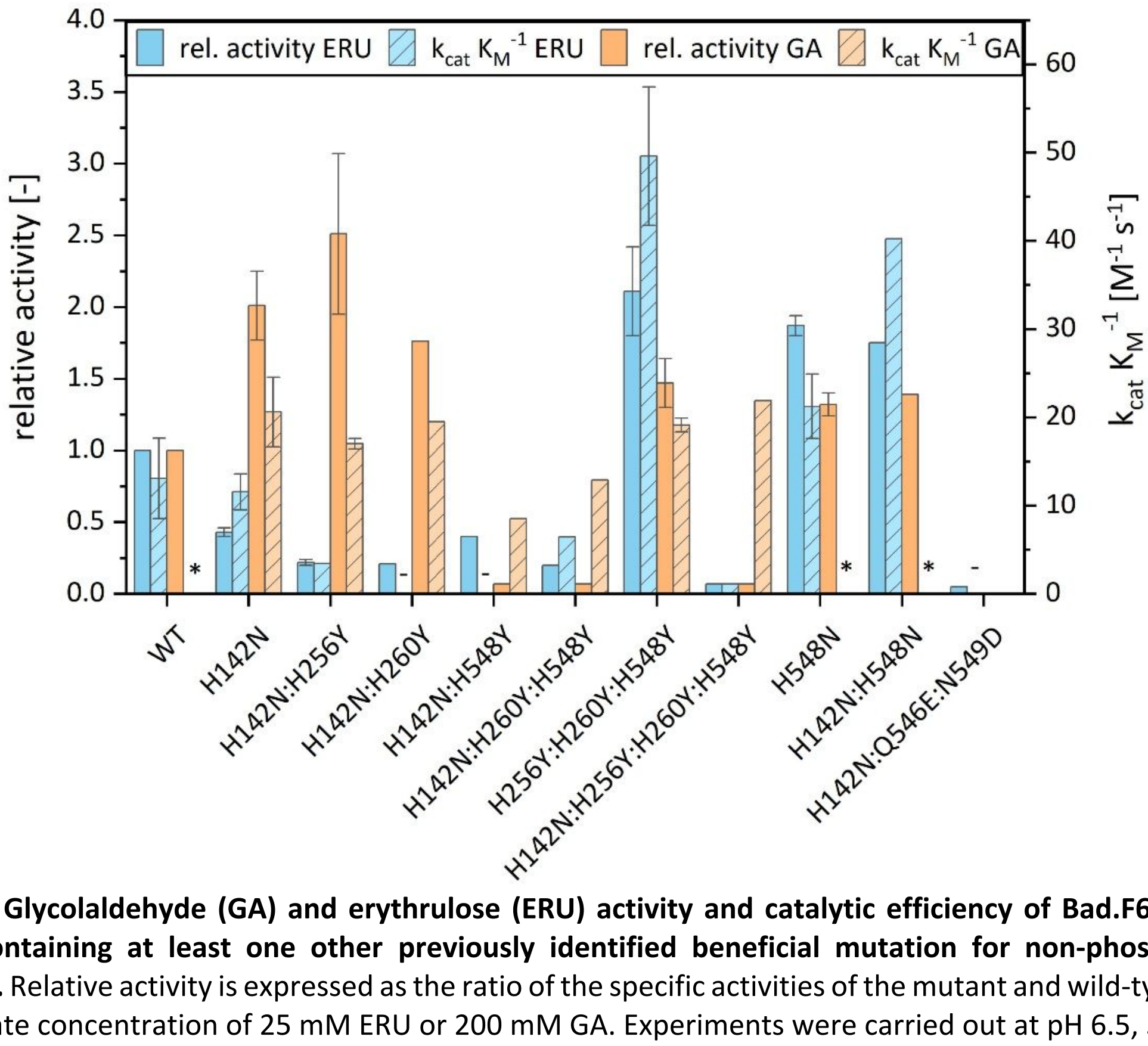

Figure S11 Glycolaldehyde (GA) and erythrulose (ERU) activity and catalytic efficiency of Bad.F6Pkt H142N variants containing at least one other previously identified beneficial mutation for non-phosphorylated substrates . Relative activity is expressed as the ratio of the specific activities of the mutant and wild-type enzyme at a substrate concentration of 25 mM ERU or 200 mM GA. Experiments were carried out at pH 6.5, 37 °C in the presence of 50 mM inorganic phosphate. Kinetic parameter fitting of experimental data to the Michaelis-Menten equation was carried out using the MATLAB R2023b curve fitting tool. In instances where substrate saturation was not observed under experimental conditions, K_M. and k_{cat} /K_M could not be determined (*). WT – wild type, (-) – not analysed. Error bars indicate deviation of the mean (n ≥ 2).

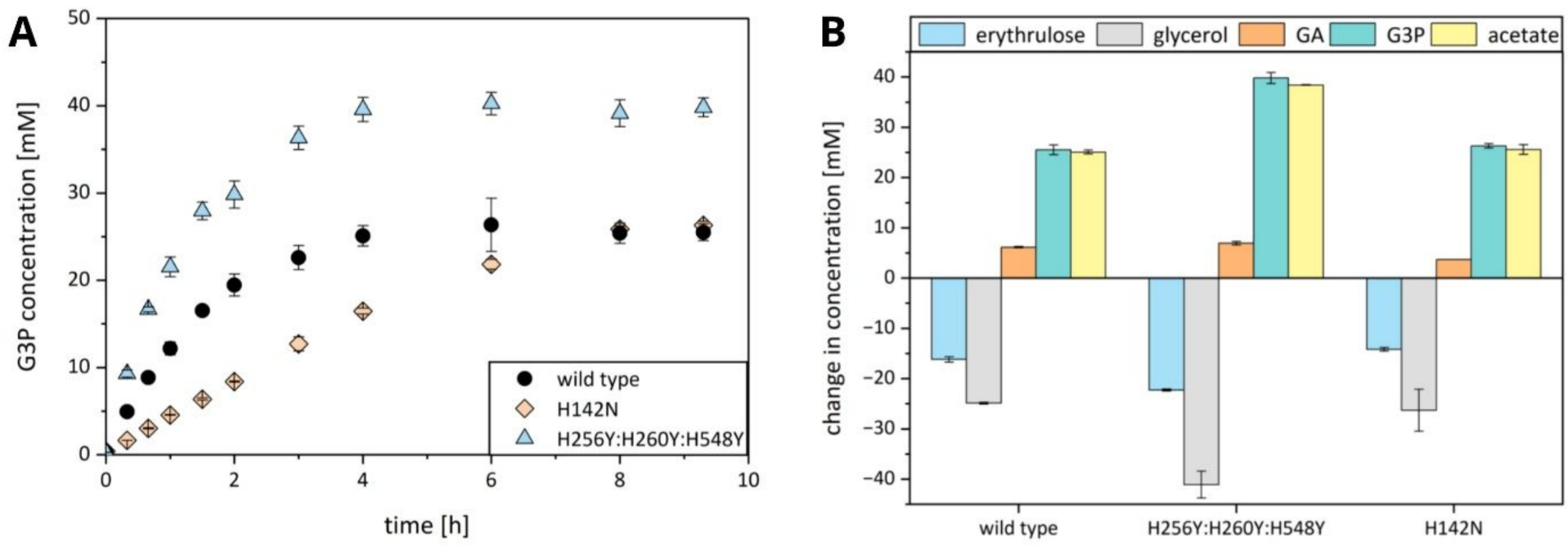

Figure S12 *In vitro* ATP regeneration from ERU by Bad.F6Pkt wild-type enzyme and different variants. A) *In vitro sn*-glycerol 3-phosphate (G3P) production with ATP regenerated from ERU **B)** changes in substrate, intermediate and product concentrations observed between the start of the reaction and the final sample after 9.5 hours. A) + B) Experiments were carried out at pH 7.0, 37 °C in the presence of 50 mM sodium phosphate and glycerol, 0.8 mM TPP, 4 mM $MgCl_2$, 1 mM ADP and a starting concentration of 25 mM D-erythrulose. Phosphoketolase activity was regulated by employing either wild-type Bad.F6Pkt or indicated variants at a concentration of 2 mg mL^{-1}. Data represent mean and deviation of biological duplicates.

Table S6 Inactivation of Bad.F6Pkt wild-type by D-threose and GA. Phosphoketolase was incubated at pH 6.5 and 37 °C in the presence of 50 mM inorganic phosphate, 4 mM $MgCl_2$ and 1 mM TPP, as well as D-threose or GA at varying concentrations. Samples were taken at regular intervals and the remaining activity was immediately determined by the hydroxamate assay. Experimental data were fitted to a first-order inactivation model [18]. Irreversible enzyme inactivation by GA was investigated by J.-O. Kundoch (unpublished results). n ≥ 2

substance	Concentration [mM]	Psuedo-First Order Rate Constant for Inactivation [h^{-1}]	Half-life [h]
D-threose	10	0.036 ± 0.005	19.7 ± 2.7
	50	0.214 ± 0.023	3.3 ± 0.4
glycolaldehyde	10	0.415 ± 0.009	1.7 ± 0.0
	45	3.923 ± 0.118	0.2 ± 0.0

References

(1) Lu, X.; Liu, Y.; Yang, Y.; Wang, S.; Wang, Q.; Wang, X.; Yan, Z.; Cheng, J.; Liu, C.; Yang, X.; Luo, H.; Yang, S.; Gou, J.; Ye, L.; Lu, L.; Zhang, Z.; Guo, Y.; Nie, Y.; Lin, J.; Li, S.; Tian, C.; Cai, T.; Zhuo, B.; Ma, H.; Wang, W.; Ma, Y.; Liu, Y.; Li, Y.; Jiang, H. Constructing a Synthetic Pathway for Acetyl-Coenzyme A from One-Carbon through Enzyme Design. *Nat. Commun.* **2019**, *10* (1378), 10. https://doi.org/10.1038/s41467-019-09095-z.

(2) Yaylayan, V. A.; Harty-Majors, S.; Ismail, A. A. Investigation of the Mechanism of Dissociation of Glycolaldehyde Dimer (2,5-Dihydroxy-1,4-Dioxane) by FTIR Spectroscopy. *Carbohydr. Res.* **1998**, *309* (1), 31–38. https://doi.org/10.1016/S0008-6215(98)00129-3.

(3) Scheidig, A. J.; Horvath, D.; Szedlacsek, S. E. Crystal Structure of a Xylulose 5-Phosphate Phosphoketolase. Insights into the Substrate Specificity for Xylulose 5-Phosphate. *J. Struct. Biol.* **2019**, *207* (1), 85–102. https://doi.org/10.1016/j.jsb.2019.04.017.

(4) Tittmann, K. Sweet Siblings with Different Faces: The Mechanisms of FBP and F6P Aldolase, Transaldolase, Transketolase and Phosphoketolase Revisited in Light of Recent Structural Data. *Bioorganic Chem.* **2014**, *57*, 263–280. https://doi.org/10.1016/j.bioorg.2014.09.001.

(5) Yevenes, A.; Frey, P. A. Cloning, Expression, Purification, Cofactor Requirements, and Steady State Kinetics of Phosphoketolase-2 from Lactobacillus Plantarum. *Bioorganic Chem.* **2008**, *36* (3), 121–127. https://doi.org/10.1016/j.bioorg.2008.03.002.

(6) Suzuki, R.; Katayama, T.; Kim, B.-J.; Wakagi, T.; Shoun, H.; Ashida, H.; Yamamoto, K.; Fushinobu, S. Crystal Structures of Phosphoketolase. *J. Biol. Chem.* **2010**, *285* (44), 34279–34287. https://doi.org/10.1074/jbc.M110.156281.

(7) Takahashi, K.; Tagami, U.; Shimba, N.; Kashiwagi, T.; Ishikawa, K.; Suzuki, E. Crystal Structure of *Bifidobacterium Longum* Phosphoketolase; Key Enzyme for Glucose Metabolism in *Bifidobacterium*. *FEBS Lett.* **2010**, *584* (18), 3855–3861. https://doi.org/10.1016/j.febslet.2010.07.043.

(8) Bayly, C. I.; Cieplak, P.; Cornell, W.; Kollman, P. A. A Well-Behaved Electrostatic Potential Based Method Using Charge Restraints for Deriving Atomic Charges: The RESP Model. *J. Phys. Chem.* **1993**, *97* (40), 10269–10280. https://doi.org/10.1021/j100142a004.

(9) Kraußer, F.; Rabe, K.; Topham, C. M.; Voland, J.; Lilienthal, L.; Kundoch, J.-O.; Ohde, D.; Liese, A.; Walther, T. Cell-Free Reaction System for ATP Regeneration from D -Fructose. *ACS Synth. Biol.* **2025**, *14* (4), 1250–1263. https://doi.org/10.1021/acssynbio.4c00877.

(10) Nakata, K.; Kashiwagi, T.; Kunishima, N.; Naitow, H.; Matsuura, Y.; Miyano, H.; Mizukoshi, T.; Tono, K.; Yabashi, M.; Nango, E.; Iwata, S. Ambient Temperature Structure of Phosphoketolase from *Bifidobacterium Longum* Determined by Serial Femtosecond X-Ray Crystallography. *Acta Crystallogr. Sect. Struct. Biol.* **2023**, *79* (4), 290–303. https://doi.org/10.1107/S2059798323001638.

(11) Hintze, B. J.; Lewis, S. M.; Richardson, J. S.; Richardson, D. C. Molprobity's Ultimate Rotamer-Library Distributions for Model Validation: MolProbity's Ultimate Rotamer-Library. *Proteins Struct. Funct. Bioinforma.* **2016**, *84* (9), 1177–1189. https://doi.org/10.1002/prot.25039.

(12) Topham, C. M.; Barbe, S.; André, I. An Atomistic Statistically Effective Energy Function for Computational Protein Design. *J. Chem. Theory Comput.* **2016**, *12* (8), 4146–4168. https://doi.org/10.1021/acs.jctc.6b00090.

(13) Topham, C. M.; Rouquier, M.; Tarrat, N.; André, I. Adaptive Smith-Waterman Residue Match Seeding for Protein Structural Alignment: Protein Structural Alignment. *Proteins Struct. Funct. Bioinforma.* **2013**, *81* (10), 1823–1839. https://doi.org/10.1002/prot.24327.

(14) Wang, L.; Li, L.; Alexov, E. P *K* a Predictions for Proteins, RNA s, and DNA s with the Gaussian Dielectric Function Using DelPhi p *K* a. *Proteins Struct. Funct. Bioinforma.* **2015**, *83* (12), 2186–2197. https://doi.org/10.1002/prot.24935.

(15) Pahari, S.; Sun, L.; Basu, S.; Alexov, E. DelPhiPKa: Including Salt in the Calculations and Enabling Polar Residues to Titrate. *Proteins Struct. Funct. Bioinforma.* **2018**, *86* (12), 1277–1283. https://doi.org/10.1002/prot.25608.

(16) Topham, C. M.; Smith, J. C. Tri-Peptide Reference Structures for the Calculation of Relative Solvent Accessible Surface Area in Protein Amino Acid Residues. *Comput. Biol. Chem.* **2015**, *54*, 33–43. https://doi.org/10.1016/j.compbiolchem.2014.11.007.
(17) Daudé, D.; Topham, C. M.; Remaud-Siméon, M.; André, I. Probing Impact of Active Site Residue Mutations on Stability and Activity of *Neisseria Polysaccharea* Amylosucrase. *Protein Sci.* **2013**, *22* (12), 1754–1765. https://doi.org/10.1002/pro.2375.
(18) Sadana, A. Enzyme Deactivation. *Biotechnol. Adv.* **1988**, *6* (3), 349-IN2. https://doi.org/10.1016/0734-9750(88)91890-3.
